# Protective role of bacillithiol in superoxide stress and Fe–S metabolism in *Bacillus subtilis*

**DOI:** 10.1002/mbo3.267

**Published:** 2015-05-18

**Authors:** Zhong Fang, Patricia C Dos Santos

**Affiliations:** Department of Chemistry, Wake Forest UniversityWinston-Salem, North Carolina, 27016

**Keywords:** Bacillithiol, dehydratase, Fe–S cluster, Suf, superoxide stress

## Abstract

Glutathione (GSH) serves as the prime thiol in most organisms as its depletion increases antibiotic and metal toxicity, impairs oxidative stress responses, and affects Fe and Fe–S cluster metabolism. Many gram-positive bacteria lack GSH, but instead produce other structurally unrelated yet functionally equivalent thiols. Among those, bacillithiol (BSH) has been recently identified in several low G+C gram-positive bacteria. In this work, we have explored the link between BSH and Fe–S metabolism in *Bacillus subtilis*. We have identified that *B. subtilis* lacking BSH is more sensitive to oxidative stress (paraquat), and metal toxicity (Cu(I) and Cd(II)), but not H_2_O_2_. Furthermore, a slow growth phenotype of BSH null strain in minimal medium was observed, which could be recovered upon the addition of selected amino acids (Leu/Ile and Glu/Gln), supplementation of iron, or chemical complementation with BSH disulfide (BSSB) to the growth medium. Interestingly, Fe–S cluster containing isopropylmalate isomerase (LeuCD) and glutamate synthase (GOGAT) showed decreased activities in BSH null strain. Deficiency of BSH also resulted in decreased levels of intracellular Fe accompanied by increased levels of manganese and altered expression levels of Fe–S cluster biosynthetic SUF components. Together, this study is the first to establish a link between BSH and Fe–S metabolism in *B. subtilis*.

## Introduction

Biothiols are involved in numerous cellular functions, including but not limited to a variety of biosynthetic pathways, detoxification by conjugation, metal metabolism, and cell division (Winterbourn and Metodiewa 1999; Jacob et al. 2006; Hider and Kong 2011). They can be classified as large molecular weight thiols, also called protein thiols, and low molecular weight (LMW) free thiols (Sen and Packer 2000). Cysteine is the most common LMW biothiol and its sulfhydryl group provides unique chemical functionality distinct from other standard amino acids. Its neutral pKa, nucleophilicity, redox properties, metal ion affinity, and bonding characteristics make Cys a versatile site for many chemical processes (Bulaj et al. 1998; Jacob et al. 2006; Roos et al. 2013).

In the late 19th century, glutathione (GSH) was identified as another abundant LMW thiol widely distributed in most cells (Rey-Pailhade 1888a,b). GSH mainly exists in its reduced form and its disulfide form (GSSG) with the cellular ratio of GSH:GSSG estimated to be around 100:1 (Fahey et al. 1978; Meister and Anderson 1983) as opposed to cysteine, whose thiol:disulfide ratio lies around 25:1 (Newton et al. 2009; Sharma et al. 2013). Early studies have shown that GSH plays a key role in a wide array of biochemical functions. First, GSH can directly serve as an antioxidant by donating electrons to reactive radicals through autoxidation reactions to form GSSG (Meister and Anderson 1983; Meister 1988; Chesney et al. 1996). Thus, under oxidative stress conditions, the GSH/GSSG ratio may fall into the 1–10 range (Reed 1990). Second, GSH serves as the substrate of biochemical reactions. For example, dehydroascorbate reductase which is a key component of the GSH-ascorbate cycle uses GSH as a substrate to produce ascorbate to detoxify H_2_O_2_ (Crook 1941). Besides the various roles of GSH on detoxifying oxidants and toxins, GSH is also involved in chemical modification of protein cysteine residues (Dalle-Donne et al. 2009). S-glutathionylation is considered a specific posttranslational modification on protein cysteine residues in response to oxidative stress or nitrosative stress (Giustarini et al. 2005; Martinez-Ruiz and Lamas 2007; Dalle-Donne et al. 2009). Several findings have also reported the vital role for S-glutathionylation in redox-regulation, energy metabolism, cellular signaling, calcium homeostasis, protein folding, and stability (Reed 1990; Cotgreave et al. 2002; Dalle-Donne et al. 2003, 2009; Pan and Berk 2007; Demasi et al. 2008; Rodriguez-Pascual et al. 2008).

Interestingly, the effect of GSH on the superoxide-sensitive Fe–S cluster-containing aconitase (ACN) of *Escherichia coli* has also been demonstrated (Gardner and Fridovich 1993a). The results showed a ∼20% decrease in ACN activity in a GSH-deficient *E. coli* strain, displaying a phenotype which could be recovered upon addition of GSH to the growth medium (Gardner and Fridovich 1993a). Earlier research indicated that the reactivation of O_2_^−^-inactivated ACN in cell extract was blocked by adding ethylenediaminetetraacetic acid (EDTA) or 2,2′-dipyridyl (DP) (Kennedy et al. 1983; Gardner and Fridovich 1992). This observation led to the proposal that the reactivation of O_2_^−^-inactivated ACN would involve the restoration of [3Fe–4S] clusters by free Fe^2+^. Overall, in this model, GSH would act as either a reductant or Fe^2+^ donor to facilitate repair of damaged Fe–S clusters. Recently, it has been reported that GSH also acts as a key component of the cytoplasmic labile iron pool providing further support for the important role of GSH in iron homeostasis (Hider and Kong 2011). Moreover, changes in the redox ratio of GSH/GSSG has been correlated with induction of manganese (Mn)-dependent superoxide dismutase expression (Gardner and Fridovich 1993b). Despite compelling evidence for GSH’s involvement in oxidative stress, thiol homeostasis, and Fe–S cluster metabolism, the complete inventory of biological processes involving GSH as well as the exact mechanism of GSH-dependent reactions is still not fully understood.

Although GSH is recognized as the dominant LMW thiol in eukaryotes and gram-negative prokaryotes, GSH is not detected in many gram-positive species. This observation suggested the existence of other LMW thiols replacing the role of GSH in those species. In fact, in Actinobacteria, mycothiol (MSH) was identified as a novel cysteine derivative (Newton et al. 1996). *Mycobacterium smegmatis* strains lacking mycothiol are more sensitive to reactive oxygen and nitrogen species, such as hydrogen peroxide, menadione, plumbagin, and *t*-butyl hydrogen peroxide (Newton et al. 1999, 2008; Rawat et al. 2002). A recent study on WhiD, a member of the WhiB-like family of Fe–S proteins, indicated that methyl mycothiol exerted a small but significant protective effect against WhiD cluster loss under oxidative stress (Crack et al. 2009).

In some low-(G+C)-content gram-positive bacteria, such as *Bacillus* species, bacillithiol (BSH) is a major LMW thiol recently discovered in bacteria lacking GSH and MSH (Newton et al. 2009). Because of the crucial roles of GSH and MSH in thiol-redox homeostasis, protein posttranslational modification, and xenobiotic detoxification, it is anticipated that some of these functions might also be performed by BSH (Helmann 2011). The BSH redox potential was characterized to be −221 mV through in vitro coupled assay with GSH/GSSG, which is higher than Cys (−223 mV) and CoASH (−234 mV) (Sharma et al. 2013). This implies that BSH does not have better capacity to buffer oxidative stress through autoxidation reaction. However, considering the low thiol *p*Ka value of BSH (*p*Ka_SH/S_^−^ = 7.97) and the relatively high concentration of BSH (0.5–5 mmol/L) (Sharma et al. 2013), it is still possible that BSH is preferable to Cys and CoA as a major redox buffer in *B. subtilis*. In addition, BSH has been shown to modulate the intracellular labile zinc pool by forming a tetrahedral BSH_2_:zinc complex with two sulfur and two oxygen ligands (Ma et al. 2014). Although BSH is not an essential metabolite under standard laboratory conditions, initial studies have identified that bacterial strains unable to synthesize this thiol displayed impaired sporulation, sensitivity to acid and salt, depleted NADPH level, and increased sensitivity to fosfomycin, hypochlorite, diamide, and methylglyoxal (Gaballa et al. 2010; Lamers et al. 2012; Roberts et al. 2013; Chandrangsu et al. 2014; Posada et al. 2014). While defects in metabolism resulting from BSH phenotypes have been identified, biochemical pathways involving BSH have not been fully explored.

The goal of this work was to investigate the role of BSH in Fe–S metabolism under normal and stress conditions. We show that lack of BSH caused a slow growth phenotype in minimal medium, which may be due to the lower activity of Fe–S enzymes involved in branched chain amino acid biosynthesis. Furthermore, growth assays under various stress conditions revealed a protective role of BSH against copper stress, superoxide stress, cadmium stress, and iron starvation. Along with the parallel Fe–S enzyme assays, thiol analysis, and intracellular metal analysis suggest the involvement of BSH in superoxide stress and metal homeostasis. Moreover, western blot analysis was performed on Fe–S cluster biosynthetic proteins in wild-type and mutant strains. Metal analysis of a BSH deletion strain under paraquat (PQ) stress also revealed a link between BSH and Mn homeostasis.

## Experimental Procedures

### Reagents

3-isopropylmalic acid and monobromobimane (mBBr) were purchased from Sigma-Aldrich (St. Louis, MO, USA). *N*-acetyl-glucosamine-malate and BSH disulfide (BSSB) were synthesized as described in Sharma et al. (2011) and Lamers et al. (2012), respectively. Primary antibodies were generated by Thermo Scientific Pierce (Waltham, MA, USA) using laboratory purified protein antigen (SufC, SufD, or MnmA) for rabbit immunization, whereas Goat anti-Rabbit IgG (H+L) with AP conjugate as secondary antibody and Lumi-Phos WB chemiluminescent substrate were both from Thermo Scientific. All the other reagents were obtained from Fisher Scientific and Sigma-Aldrich.

### Bacterial growth

*Bacillus subtilis* CU1065 (wt), HB11002 (*ΔbshA),* HB110079 *(ΔbshC*), and HB110091 (*ΔbshC amyE*::P_xylA_*bshC*) strains were kindly provided by Prof. John Helmann (Cornell University) (Gaballa et al. 2010). Strains were outgrown either in Luria broth (LB) medium or Spizizen’s minimal medium plus 10 *μ*g/mL tryptophan (MM). For the growth experiments, supplementary components were added where appropriate to the following concentrations: casamino acid, 0.05%; l-glutamine, 120 *μ*g/mL; l-glutamate, 120 *μ*g/mL; l-leucine, 46 *μ*g/mL; l-isoleucine, 34 *μ*g/mL. When antibiotics were needed, medium was supplemented with 12.5 *μ*g/mL erythromycin and 12.5 *μ*g/mL lincomycin or 40 *μ*g/mL kanamycin. In growth assays, strains cultured up to OD_600_ of 1.0 (measured using a 1 cm cuvette) in MM were diluted to OD_600_ of 0.05 into 200 *μ*L of fresh medium and allowed to grow at 37°C on Falcon 96-well plate. The cell growth rate was monitored at Abs_600_ using Synergy H1 plate reader from BioTek (Winooski, VT, USA).

### Enzymatic assays

Activities of Fe–S cluster containing proteins, mononuclear iron enzymes and the non-Fe–S cluster containing malate dehydrogenase (MDH) were determined in clear cell-free lysate. *Bacillus subtilis* wild-type and *ΔbshA* were grown in MM to OD_600_ of 0.8–1.0. Cells were harvested by centrifugation at 5000*g* for 10 min and frozen in −80°C until use. Cell pellets from 500 mL cultures were transferred into anaerobic glove box and washed by 5 mL of 25 mmol/L Tris-HCl, 150 mmol/L NaCl, 10% glycerol, pH 8.0 degassed buffer. The resulting pellets were then resuspended in 2 mL buffer and disrupted by sonication anaerobically. Clear cell extracts were collected by centrifugation at 13,200*g* for 10 min and stored in sealed vials for assays. Protein concentrations were determined by Bradford protein assay using BSA as standard.

Glutamate synthase (GOGAT) assays were conducted using the protocol as described in reference (Outten et al. 2004) with minor modifications. To ensure consistent reaction conditions, 200 mmol/L Tris-HCl (pH 8.0), 0.1 mol/L buffered *α*-ketoglutarate, and 8 mmol/L NADPH were mixed in a ratio of 32:2:1 to make a stock reaction mix. In each assay, 875 *μ*L of fresh stock reaction mix and 75 *μ*L of clear cell lysate were injected into a sealed cuvette filled with argon gas. The decreasing slope of absorbance at 340 nm (NADPH oxidation) was recorded as S1, and then 50 *μ*L of 0.2 mol/L l-glutamine was added and second slope was recorded as S2. The specific activity of GOGAT was calculated by the following equation:

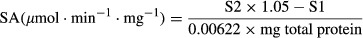


Isopropylmalate isomerase (LeuCD) was assayed by using 3-isopropylmalic acid as substrate and by following the formation of the intermediate dimethylcitraconate at 235 nm (Fultz and Kemper 1981; Manikandan et al. 2011). In brief, 800 *μ*L of 200 mmol/L phosphate buffer (pH 7.0) and 100 *μ*L of cell extract were mixed first, followed by addition of 100 *μ*L of 10 mmol/L 3-isopropylmalic acid to start the reaction. Blanks were carried out in the absence of substrate, and extinction coefficient of 4.668 mmol L^−1^ cm^−1^ was used to calculate the activity.

ACN catalyzes a reversible reaction between citrate and iso-citrate through an intermediate cis-aconitate which has absorption at 240 nm (Alen and Sonenshein 1999). Each assay reaction contained 700 *μ*L of 100 mmol/L Tris-HCl, pH 8.0, 50 *μ*L of cell extract and 100 *μ*L of 200 mmol/L Tris-Na-Citrate. The increase in absorbance at 240 nm was measured and a millimolar extinction coefficient of 3.4 mmol L^−1^ cm^−1^ was used to calculate the activities. Control reactions were performed without substrate.

The activity of MDH was measured by following the consumption of NADH at 340 nm (Siegel 1969). The reaction mix contained 50 *μ*mol of Tris-HCl (pH 7.4), 0.38 *μ*mol of oxaloacetate, 0.15 *μ*mol NADH and 50 *μ*L of cell extract in a total volume of 1 mL. The decrease in absorption at 340 nm was measured over time.

Quercetin 2,3-dioxygenase (QD) catalyzes the cleavage of the O-heteroaromatic ring of quercetin to produce 2-protocatechuoylphloroglucinol carboxylic acid and carbon monoxide. QD activity was measured based on the decomposition of quercetin as indicated by the decrease in absorption at 367 nm (Bowater et al. 2004). Cell lysate was prepared anaerobically as described above and by adding 5 *μ*mol/L quercetin in lysis buffer to stabilize metal cofactor. A typical assay reaction contained 1 mL of 200 mmol/L Tris-HCl (pH, 8.0), 100 *μ*L of crude extract and 50 *μ*L of 1.2 mmol/L quercetin in dimethyl sulfoxide. The absorption at 367 nm was monitored over time for 2 min and an extinction coefficient of 20 mmol/L^−1^ cm^−1^ was used to calculate the activity.

Assays of threonine dehydrogenase (Tdh) were performed by following the previous reported protocol (Gu and Imlay 2013). The cell lysate was prepared anaerobically using Tris-HCl buffer plus 5 mmol/L l-threonine. In each assay, 600 *μ*L of 50 mmol/L Bicine (pH, 8.5), 100 *μ*L of cell extract and 20 *μ*L of 50 mmol/L NAD were mixed in a sealed cuvette, and then 300 *μ*L of buffered 90 mmol/L l-threonine was added to initiate the reaction. The activity of Tdh was measured by monitoring the production of NADH at Abs_340_.

### ICP-OES analysis for cellular metal concentration

*Bacillus subtilis* wild-type and *ΔbshA* strains were grown in MM until OD_600_ reached 1.0. Culture aliquots (300 mL) were removed before the addition of 100 *μ*mol/L PQ, and 20 min, 50 min after stress exposure. Cells were harvested by centrifugation and washed twice with 10 mL of chilled 25 mmol/L phosphate buffer (pH.7.4) containing 1 mmol/L EDTA. The resulting cell pellets were immediately washed twice with the same buffer without EDTA and resuspended with 10 mL of phosphate buffer. Aliquots of resuspended cells (2 mL) were transferred to an eppendorf tube, in which 20 *μ*L of 20 mg/mL lysozyme was added. The sample was incubated at 37°C for 30 min, followed by adding 2 mL of 5% nitric acid with 0.1% (v/w) Triton X-100. The sample was digested at 95°C for 30 min and supernatant was collected by centrifugation. Each supernatant was diluted with an equal volume of deionized water for the measurement of total metal content.

The concentrations of labile metal (defined as metals not associated with a higher molecular weight fraction, <3000 Da) were quantified as follow. Eight milliliters of cell suspension from above was disrupted by an Avestin EmulsiFlex-C5 homogenizer anaerobically, and centrifuged at 13,200*g* for 20 min. Supernatant was transferred and adjusted to 8 mL with degassed phosphate buffer. Cell-free extract (2 mL) was mixed with 2 mL of 5% nitric acid containing 0.1% (v/w) Triton X-100, followed by incubation and dilution as described above. The resulting sample contained both labile metal and protein-associated metal. The remaining 6 mL of supernatant was transferred to an Amicon Ultra-15 centrifugal tube with 3000 MW cutoff, and spun at 5000*g*. Two milliliter of flow-through was applied to prepare the sample containing labile metal by following the same procedure as described above.

All the samples were analyzed using inductively coupled plasma optical emission spectrometry (ICP-OES) from Teledyne Leeman Labs (Hudson, NH, USA). The wavelengths for the detection of each metal were as follows (nm): Fe, 238.204, 239.563; Mn, 257.610, 259.372; Sample concentrations were calculated using metal standard solutions and normalized to the dry weights (DWs) of cell pellets.

### Quantification of thiol levels under stress conditions

The contents of reduced thiol BSH, oxidized thiol BSSB and protein thiol (BSSProt) were prepared as described by Newton et al. (2009), Chi et al. (2013). In brief, 25 mL of cultures in MM were challenged with stress at OD_600_ of 1.0 and harvested at 0, 20, and 50 min after exposure. Controls were prepared by adding 5 mmol/L of *N*-ethylmaleimide (NEM) to block-free thiol, and followed by derivatization with 2.5 mmol/L of mBBr. The preparations of disulfide samples were similar to controls, except being reduced with dithiothreitol (DTT) prior derivatization. Reduced thiol was prepared by direct incubation with 2 mmol/L mBBr. Total thiol content was measured by incubating cell pellet with 500 *μ*L of 25 mmol/L 4-(2-hydroxyl)-1-piperazineethanesulfonic acid (HEPES), 2 mmol/L DTT, pH 8.0 on ice for 1 h, followed by adding 500 *μ*L of 8 mmol/L mBBr in acetonitrile. The mixture was incubated at 65°C for 20 min and quenched by the addition of 10 *μ*L of 2 N HCl. All samples were diluted five times with 5 mmol/L HCl and injected 50 *μ*L for high performance liquid chromatography (HPLC) analysis. The HPLC method was conducted with a Waters Symmetry C8 column (3.9 × 150 mm, 5 *μ*m) using the gradient as described in (Newton et al. 2009). The intracellular concentrations of BSH derivatives were calculated based on BSH standard and normalized to cell DW.

### Western blot analysis

Cells were grown in MM to OD_600_ of 0.8–1.0 and then challenged with different stress for 30 min. Cultures were harvested by centrifugation and stored in −20°C. Cell pellets were resuspended and disrupted by sonication, the supernatant containing soluble protein extract was then quantified using the Bradford assay (Biorad, Hercules, CA, USA). For sodium dodecyl sulfate polyacrylamide gel electrophoresis (SDS-PAGE), 50 *μ*g of total protein was loaded into each well and the gel was subjected to western blot analysis. Custom primary antibodies were generated by Thermo scientific through rabbit immunization with heterologously expressed, purified *B. subtilis* SufC, SufB, and MnmA. The western blot membrane was incubated with each primary antibody, secondary antibody followed by Lumi-Phos WB chemiluminescent substrate, and then exposed to Carestream® Kodak film from Fisher to fix the protein bands. The films were then been scanned and images were processed by ImageJ software (Bethesda, MD, USA), each band area was quantified (Gassmann et al. 2009). The amount of protein in terms of *μ*g was calculated based on a standard curve from pure proteins in the range 0.02–0.1 *μ*g.

## Results

### BSH null strains show a slow growth phenotype in minimal medium

**Figure 1:**
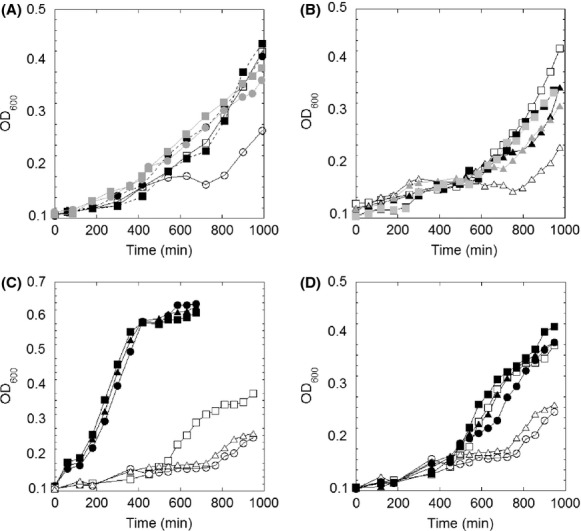
Growth profile of *Bacillus subtilis* strain lacking BSH in MM. (A) Growth curves of wild-type *B. subtilis* (square) and *ΔbshA* strain (circle) in Spizizen’s MM (empty), MM plus 200 *μ*mol/L of *N*-acetyl-glucosamine-malate (gray) or MM plus 200 *μ*mol/L of BSSB (black). (B) Growth curves of wild type (square), *ΔbshC amyE:P*_*xylA*_*bshC* (triangle) in MM (empty), MM plus 2% xylose (black) and MM plus 200 *μ*mol/L of BSSB (gray). (C) Growth curves of wild type (square), *ΔbshA* (circle), and *ΔbshC amyE:P*_*xylA*_*bshC* (triangle) strains in MM (empty) and in MM plus 0.5% casamino acid (black). (D) Growth curves in the presence (black) and absence (empty) of 50 *μ*mol/L of Fe^2+^ for wild type (square) and *ΔbshA* strain (circle). The curves shown are representatives of at least three independent experiments.

The biosynthesis of BSH was proposed to be a three-step reaction, utilizing BshA, BshB, and BshC enzymes. BshA catalyzes the ligation of L-malate onto the carbon 1 position of UDP-*N*-acetyl-glucosamine to release UDP molecule (Parsonage et al. 2010). BshB is a metal-dependent deacetylase which cleaves the amide bond of *N*-acetyl-glucosamine-malate to form *N*-glucosamine-malate (Gaballa et al. 2010; Fang et al. 2013). The last step of BSH biosynthesis was suggested to be the condensation reaction of l-Cys and *N*-glucosamine-malate (Helmann 2011). Inactivation of *bshA* or *bshC* completely depleted intracellular BSH, whereas deletion of *bshB* only caused a modest decrease in BSH levels. Initial studies showed that inactivation of BSH biosynthetic genes caused no effects on *B. subtilis* growth rates in LB-rich medium (Gaballa et al. 2010). This observation suggested that the depletion of BSH did not cause major defects in metabolism. However, it was also possible that the components in LB medium replaced the role of BSH and concealed phenotypes in mutant strains. To rule out this possibility, *B. subtilis* wild-type and mutant strains were grown in Spizizen’s minimal medium (MM). Interestingly, unlike the similar growth profiles in LB (Fig. S1), the *ΔbshA* strain displayed a modest, yet-reproducible slower growth rate in MM as shown in Figure1A. As *bshA* is located in an operon along with ten other coding sequences including *bshB*, the slow growth phenotype could have been attributed to a polar effect on downstream genes. We have addressed this potential concern in three experiments. First, the addition of enzymatically produced *N*-acetyl-glucosamine-malate (GlcNAc-malate) (Fang et al. 2013), the product of the BshA reaction, to cultures of *ΔbshA* strain recovered the phenotype in MM (Figure1A). Second, the growth rate of a strain containing a deletion within another biosynthetic gene, *ΔbshC* strain, showed a similar growth profile in MM (Figure1B). In *B. subtilis*, *bshC* is a monocystronic gene located remotely from other *bshA* and *bshB* biosynthetic genes. Third, addition of BSSB, the oxidized form of chemically synthesized BSH, to either *ΔbshA* or *ΔbshC cultures* was able to repair these growth defects (Fig.1A and B). Interestingly, addition of cystine or GSSG did not rescue the phenotype associated with lack of BSH. Collectively, these results confirmed that phenotypes associated with *ΔbshA* strain were attributed to the absence of BSH.

Further studies revealed that the growth phenotype was fully restored with the supplement of 0.05% casamino acid (Fig.1C). Via the screening of different combinations of amino acids, it was concluded that l-glutamine, l-glutamate and branched-chain amino acids l-leucine and l-isoleucine were able to correct the growth defect of the BSH-deletion strain (Fig. S1). Interestingly, the addition of 50 *μ*mol/L Fe^2+^ also restored growth defects associated with lack of BSH in MM (Fig.1D), whereas other divalent metals (Mn, Mg, Ca, and Zn) showed no drastic restoring effect (data not shown), confirming the specificity of Fe.

### The depletion of BSH decreases the activities of Fe–S enzymes

**Figure 2:**
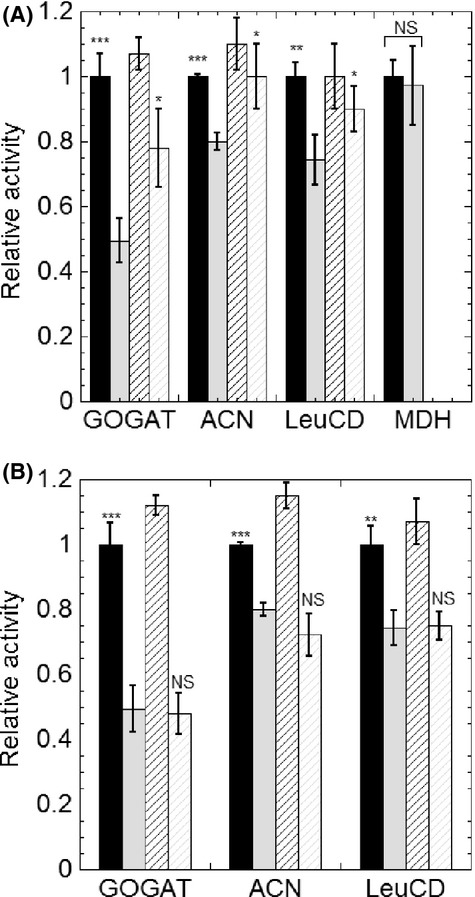
Lack of BSH leads to lower activity levels of Fe–S enzymes. (A) Activities of the Fe–S proteins glutamate synthase (GOGAT), aconitase (ACN), isopropyl malate isomerase (LeuCD) and the non-Fe–S protein malate dehydrogenase (MDH) in cell lysates of *Bacillus subtilis* wild type (black) and *ΔbshA* strain (gray) cultured in MM (filled bar) or MM containing an additional 50 *μ*mol/L of Fe^2+^ (diagonal striped bar). (B) *Bacillus subtilis* wild-type strain (black) and *ΔbshA* strain (gray) cultured MM and the activity of Fe–S enzymes was quantified before (filled bar) and after the addition of 50 *μ*mol/L of Fe^2+^ to cell lysates (upright striped bar). The activities of GOGAT, ACN, LeuCD, and MDH for wild-type *B. subtilis* were measured to be 40, 131, 17, and 1051 (nmol/min per mg total protein in crude extract), respectively. The activities of the *ΔbshA* strain were normalized to the activities of wild type in MM. All assays were performed in triplicates. The statistical analysis was performed using unpaired *t* test, *P* values were obtained by comparing the *ΔbshA* without iron to wild-type w/o Fe and to *ΔbshA* with Fe. Comparisons of wild-type activities with and without Fe in both panels were not statistical significant. (NS, not significant, *P <* *0.05, **P <* *0.01, ***P <* *0.001).

Initial growth experiments suggested the involvement of BSH in the synthesis of selected amino acids (leucine, isoleucine, glutamine, and glutamate). The biosynthetic pathways of these amino acids include Fe–S enzymes. Glutamine oxoglutarate aminotransferase (GOGAT) containing two 4Fe–4S clusters and one 3Fe–4S cluster catalyzes the reversible conversion from l-glutamine and *α*-ketoglutarate to l-glutamate. LeuCD, a dihydroxy-acid dehydratase in the biosynthesis of l-isoleucine, requires [4Fe–4S] cluster for its function. Likewise, the 4Fe–4S enzyme ACN in TCA cycle also indirectly contributes to synthesis of amino acids. The *E. coli* GSH null strain displayed a decrease in ACN activity (80% of WT levels) under standard growth conditions (Gardner and Fridovich 1993a). Therefore, we hypothesized that the growth defects of the *ΔbshA* strain were caused by decreased Fe–S enzyme activities. To test this hypothesis, the specific activities of GOGAT, LeuCD, and ACN were determined in cell lysates from cultures harvested at mid-log phase (OD_600_ of 0.8–1.0 when using 1 cm cuvette). The activ-ity levels of these enzymes in the wild-type cell lysates were comparable to the previously reported activities (Bohannon and Sonenshein 1989; Alen and Sonenshein 1999), and modestly reduced in the strain lacking BSH (Fig.2A). *Bacillus subtilis* genome encodes for one copy of ACN which shows 51% sequence identity of the *E. coli* AcnA, which is the oxygen-resistant isoform of ACN. As a control, the activity of MDH, a non-Fe/S enzyme, was not significantly different in either strain. Supplementation of growth cultures with 50 *μ*mol/L Fe^2+^, a condition identified to suppress BSH^−^ phenotype in MM, led to an increase in GOGAT, ACN, and LeuCD activities in *ΔbshA* strain by 28%, 17%, and 16%, respectively, whereas causing no effect to the levels detected for the wild-type cell lysates (Fig.2A). Similar results were also reported in *Saccharomyces cerevisiae Δgsh1* strain, in which the growth defect from deletion of GSH was also repaired upon addition of Fe^3+^ in the medium, and the LeuCD activity was also enhanced after 1-h exposure to Fe^3+^ (Kumar et al. 2011). In *B. subtilis* cultures, however, posthumous ex vivo addition of 50 *μ*mol/L of Fe^2+^ in the cell lysates did not improved the activity levels of GOGAT, ACN, and LeuCD in both the wild-type and *ΔbshA* strains (Fig.2B).

In addition to Fe–S enzymes, mononuclear iron enzymes are also known to be susceptible to changes in the cellular Fe status and found to respond to reactive oxygen species (Anjem and Imlay 2012). In this study, we determined the activity of two of these metalloenzymes, QD and Tdh. The lack of BSH caused a slight decrease in their activities (Fig. S2). The addition of exogenous iron into the cell lysates did not enhance the activities, which confirmed the preparation of cell lysate was strictly anoxic.

### BSH participates in metal stress response

**Figure 3:**
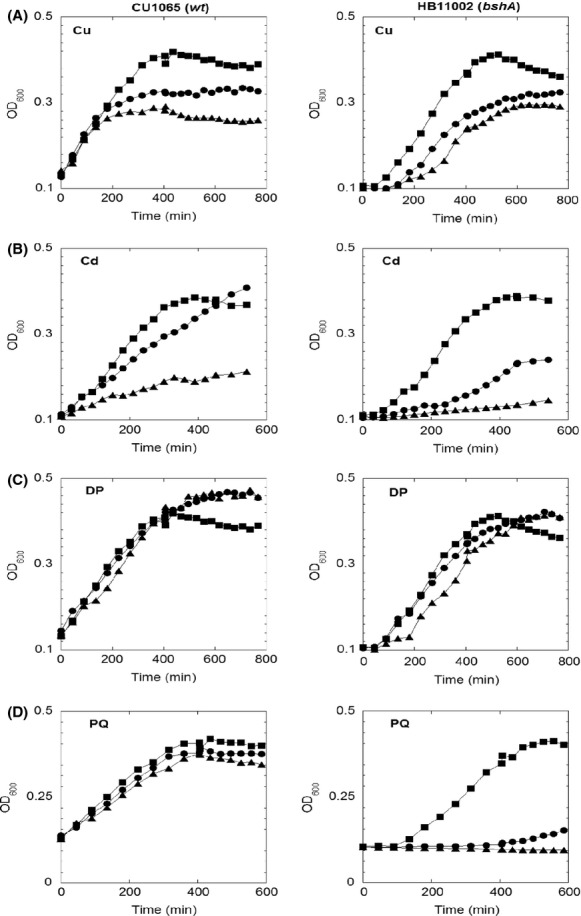
Growth curves of wild-type *Bacillus subtilis* (left panel) and *ΔbshA* strain (right panel) in MM with 0.05% casamino acid in presence of various stress challenges. (A) Cultures contained 0 *μ*mol/L (square), 100 *μ*mol/L (circle), and 300 *μ*mol/L (triangle) of CuCl; (B) Cultures contained 0 *μ*mol/L (square), 20 *μ*mol/L (circle), and 50 *μ*mol/L (triangle) of CdCl_2_. (C) Cultures contained 0 *μ*mol/L (square), 100 *μ*mol/L (circle), and 200 *μ*mol/L (triangle) of 2,2′-dypridyl. (D) Cultures contained 0 *μ*mol/L (square), 100 *μ*mol/L (circle), and 250 *μ*mol/L (triangle) of paraquat. The curves shown are representatives of at least three independent experiments.

The effects of oxidative stress on the *B. subtilis ΔbshA* strain have been previously investigated using zone inhibition assays demonstrating that both wild-type and BSH^−^ strain had the same sensitivity to H_2_O_2_ (Gaballa et al. 2010). Herein, the role of BSH against a wide range of stresses was investigated in MM supplemented with casamino acids. This growth medium not only ensured similar growth rates for both strains under no stress condition (Figs.1C, 3), but also eliminated the interference from potential protective biomolecules present in rich medium. Initial screens involved growth assays in presence of 0–300 *μ*mol/L H_2_O_2_ (data not shown), which showed no differences between wild-type and *ΔbshA* strains, in agreement with a previous report (Gaballa et al. 2010). Given the involvement of GSH in metal homeostasis, Fe–S biogenesis, and bacterial response to environmental stressors, we investigated the growth profiles of both *B. subtilis* wild-type and *bshA* deletion strains under conditions known to destabilize metal homeostasis and Fe–S metabolism such as copper (Cu(I)), cadmium (Cd(II)), as well Fe starvation conditions.

**Figure 4:**
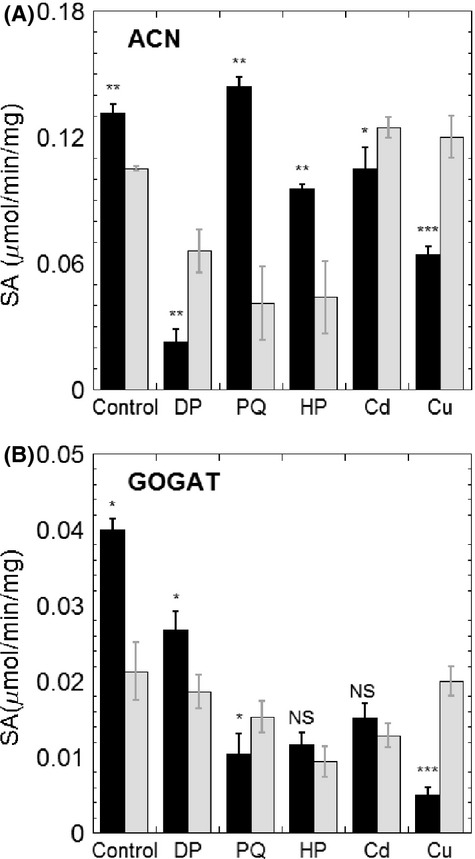
Specific activities of ACN (A) and GOGAT (B) under stress conditions. Cells were grown in MM to late log phase (OD_600_ of 0.8–1.0 measured with a 1 cm cuvette), at 37°C, and then 100 *μ*mol/L of 2,2′-dypridyl (DP), 100 *μ*mol/L of paraquat (PQ), 100 *μ*mol/L of H_2_O_2_ (HP), 50 *μ*mol/L of CdCl_2_ (Cd), or 100 *μ*mol/L of CuCl (Cu) was added to challenge the cultures for 30 min. Cell lysates were prepared as described in Experimental Procedures. The wild-type strain is represented in black bars, whereas *ΔbshA* strain is shown in gray bars. All assays were repeated in triplicates. The statistical analysis was performed using unpaired *t* test, *P* values compare *ΔbshA* sample with wild-type sample (NS, not significant, *P <* *0.05, **P <* *0.01, ***P <* *0.001).

**Figure 5:**
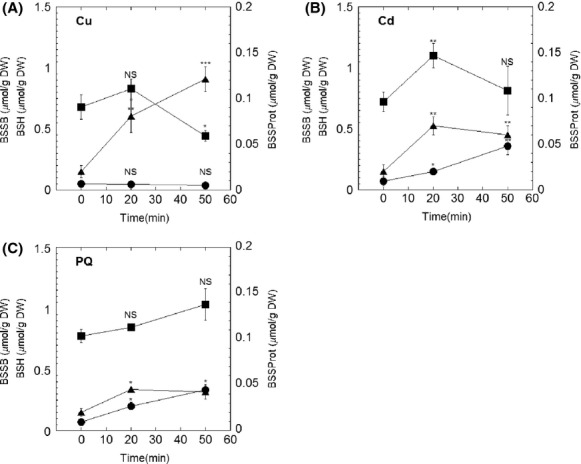
Cellular BSH redox status upon metal and superoxide stress. Intracellular levels of BSH (square), BSSB (circle), and BSSProt (triangle) were determined in *Bacillus subtilis* wild-type cells cultured in MM before (time 0) and after challenge with 100 *μ*mol/L of Cu (I) (A), 50 *μ*mol/L of Cd (II) (B) or 100 *μ*mol/L of paraquat (C). Oxidized BSH was determined when associated to intracellular small molecular weight thiol (BSSB) or large-molecule thiol (BSSProt) as described in the Experimental Procedures. The statistical analysis was performed using unpaired *t* test, *P* values compare time 0 sample with other samples (NS, not significant, *P <* *0.05, **P <* *0.01, ***P <* *0.001).

Cu(I) can strongly trigger Fenton-like reactions to produce reactive hydroxyl radicals, which further cause a global oxidative stress response, such as decreased activities for Fe–S enzymes (Macomber and Imlay 2009). As shown in Figure3A, the addition of Cu(I) did not inhibit the initial growth rate of the wild-type strain, but decreased the OD_600_ in stationary phase from 0.4 to 0.265, displaying similar growth inhibition as previously reported for *B. subtilis* (Chillappagari et al. 2010). The BSH mutant, however, showed a concentration-dependent inhibition of the initial growth rate, which indicated the protective role of BSH against Cu(I) stress. The addition of Cu(I) slightly decreased the concentration of BSH (from 0.68 to 0.44 *μ*mol/g DW) and BSSB (from 0.052 to 0.035 *μ*mol/g DW), but it significantly stimulated protein S-bacillithiolation by sixfold (from 0.02 to 0.12 *μ*mol/g DW) (Fig.5A). Wild-type cells when cultured in MM and challenged with Cu(I) for 30 min showed reduction of 50% and 87% of ACN and GOGAT activities (Fig.4). This supported previous proposals that Fe–S enzymes were the targets for Cu(I) stress in *E. coli* (Macomber and Imlay 2009) and *B. subtilis* (Macomber and Imlay 2009; Chillappagari et al. 2010). However, in the mutant strain the activities of these enzymes were not further affected with Cu(I) challenge (Fig.4).

Cadmium ion (Cd(II)) has a high-binding affinity for sulfur (Nies 2007). Therefore, the toxicity of Cd(II) has been suggested to be the result of the binding of Cd(II) to free thiols, protein thiols, Fe–S centers and other sulfur-rich compounds (Helbig et al. 2008). Cd(II) challenge affected the growth profile of both wild-type and mutant strains, whereas displaying a more dramatic effect on the growth rate of the strain lacking BSH (Fig.3B). Thiol analysis showed that despite the levels of BSH remaining at ∼0.8 *μ*mol/g DW within 50-min treatment, the BSSB content increased sevenfold, from 0.07 *μ*mol/g DW to 0.35 *μ*mol/g DW (Fig.5B). The activities of Fe–S enzymes were also determined in cell lysates of cultures subjected to 50 *μ*mol/L Cd(II) challenge for 30 min. While the addition of Cd(II) to wild-type cultures decreased ACN by only 20% (Fig.4A), GOGAT was inactivated to nearly half of its initial activity (Fig.4B). Similar to the effect observed for Cu(I) stress, the addition of Cd(II) did not cause further damage to the activity of these Fe–S enzymes in BSH null strain. Interestingly, deletion of BSH caused a 20% decrease in intracellular zinc which is a substrate for the CadA resistance system (Tsai et al. 1992; Ma et al. 2014). As a result, the CadA efflux ATPase was repressed and more Cd(II) was accumulated inside cells, which may explain the observed elevated sensitivity of BSH null strain to Cd(II).

As exogenous addition of iron to cultures of *B. subtilis ΔbshA* recovered growth defects in MM and partially corrected biochemical defects associated with the loss of BSH, we sought to determine the effects of iron starvation in both strains. The growth profile of wild-type and mutant were examined under iron starvation conditions in the presence of different concentrations of iron chelator DP in MM supplemented with casamino acids. Only a minor inhibitory effect was observed in the mutant strain with 200 *μ*mol/L DP (Fig.3C). Nonetheless, DP challenge elicited response similar to what has been observed for Cu(I) and Cd(II) stresses (Fig.4); that is, the addition of DP to growth medium resulted in 80% and 40% decrease in ACN and GOGAT activities in wild-type extracts, whereas causing modest to no inhibition in cells lacking BSH.

### *Bacillus subtilis* strains lacking BSH are sensitive to superoxide stress

**Figure 6:**
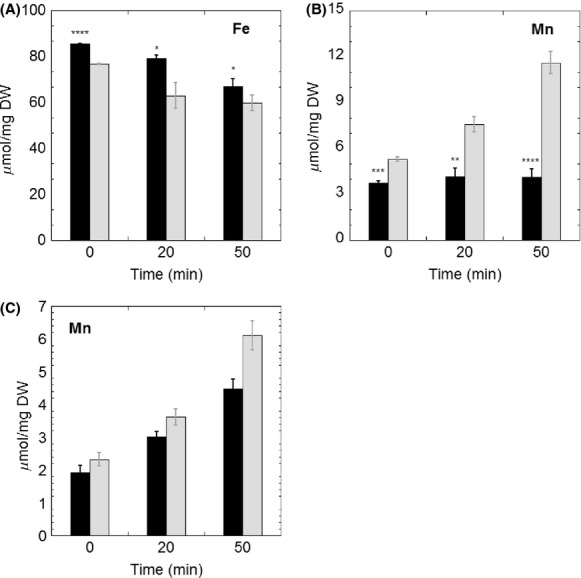
Total iron (A) and manganese (B) concentration analysis in wild-type *Bacillus subtilis* (black bar) and *ΔbshA* strain (gray bar) after exposure to 100 *μ*mol/L of paraquat stress. All measurements were performed in triplicate from independent cultures. The statistical analysis was performed using unpaired *t* test, *P* values compare *ΔbshA* sample with wild-type sample (NS, not significant, *P <* *0.05, **P <* *0.01, ***P <* *0.001, ****P <* *0.0001). (C) Manganese distribution in *ΔbshA* strain under paraquat stress as shown labile (black bar) and protein-associated manganese (gray bar).

Lastly, these strains were challenged with PQ, which can induce superoxide stress in vivo. When treated with 250 *μ*mol/L of PQ, the mutant strain showed a remarkable growth reduction, whereas wild type remained unaffected (Fig.3D). Thiol analysis also indicated that the content of BSSB increased by near fivefold (from 0.07 to 0.34 *μ*mol/g DW) and protein-associated BSH increased by twofold (from 0.02 to 0.04 *μ*mol/g DW) (Fig.5C). PQ challenge has also been associated with iron starvation and NADPH depletion in *B. anthracis* (Pohl et al. 2011). Therefore, the total Fe content of cells cultured in the presence of PQ was determined. The mutant strain displayed small decrease in the total Fe when compared with the wild type in the absence of PQ stressor (Fig.6A). In both wild-type and *ΔbshA* strains, PQ challenge led to modest decrease in the total iron accompanied by minor increase in the Fe content associated to the LMW fraction (labile iron pool). In the presence of 100 *μ*mol/L of PQ, the activity of GOGAT was more affected in the wild type than in the *ΔbshA* strain (Fig.4B). The ACN activity, on the other hand, remained unchanged in the wild-type strain, whereas displaying a nearly 50% decrease in cells extracts lacking BSH (Fig.4A). Collectively, the PQ sensitivity associated with lack of BSH along with the thiol analysis of wild-type cultures exposed to PQ provides evidence for a protective role of BSH against superoxide stress.

### BSH deficiency leads to Mn uptake under superoxide stress

Oxidative stress has also been shown to trigger the import of Mn and induction of Mn-dependent superoxide dismutase. In *E. coli*, the expression of manganese superoxide dismutase (MnSOD) is positively correlated with the redox ratio of GSH/GSSG (Gardner and Fridovich 1993b). The partial cross-regulation between thiol-redox homeostasis and Fe/Mn levels (Helmann 2014), led us to determine whether the lack of BSH would affect cellular accumulation of these metals. Intracellular content of Fe and Mn levels were determined in wild-type and *ΔbshA* cells cultured in MM (Fig.6A and B). While the lack of BSH did not cause drastic changes in the levels of Fe under stress conditions, PQ challenge elicited a time-dependent increase in intracellular Mn content for *ΔbshA* cells. As shown in Figure6B, the Mn level in the wild type remained unaltered under PQ stress, whereas the concentration of Mn in the mutant increased by more than twofold over a 50-min treatment. This observation followed a similar response observed under oxidative challenge in *E. coli*, in which deletion of catalase and peroxidase introduced oxidative stress that led to further increase in intracellular Mn concentration (Anjem et al. 2009). Based on phenotypic and biochemical analysis in *E. coli*, it has been proposed that Mn could protect peroxide-stressed cells by replacing Fe in mononuclear enzymes and by decreasing levels of Fe-triggered Fenton reaction (Anjem et al. 2009). In agreement with this model, PQ challenge led to increased levels of Mn associated with proteins, supporting a protection mechanism of Mn in *B. subtilis* (Fig.6C). In addition, the activity of MnSOD was determined in cell lysates of wild-type and *ΔbshA* strains before and 30 min after 100 *μ*mol/L PQ challenge. As shown in Figure S3, the MnSOD activity in *ΔbshA* of 15.2 ± 1.3 unit/min per mg was higher than activity detected for the wild-type strain (11.8 ± 1.0 units/min per mg). This suggests that the deletion of BSH caused a modest increase in MnSOD even under standard conditions (i.e., when cells were cultured in MM). PQ treatment caused no significant change to MnSOD activity levels in wild type, but it led to a 1.5-fold increase in the strain lacking BSH (from 15.2 ± 1.3 to 23.3 ± 1.0 units/min per mg). Activation of MnSOD elicited by PQ stress in the *ΔbshA* strain provided additional evidence for the increase levels of protein-associated Mn determined through ICP-OES analysis. Besides the protective role of Mn mentioned above, early studies in *B. subtilis* suggested that free Mn was also capable of eliminating the superoxide stress through an unknown mechanism (Inaoka et al. 1999). Therefore, ICP-OES analysis was also carried out for free Mn or that associated to LMW fraction Mn (Fig.6C). Levels of free Mn showed a similar increasing trend as the protein-associated Mn in response to PQ stress. These observations suggested a combined protection mechanism of Mn through metallating enzymes and increasing LMW Mn pool under PQ stress.

### BSH and Fe–S biosynthesis

**Figure 7:**
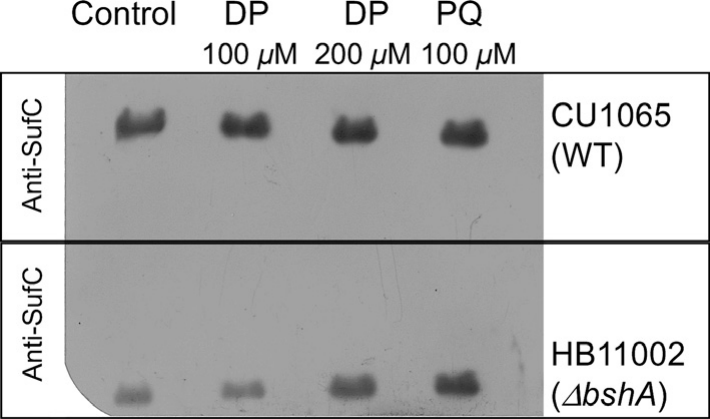
Western blot analysis of Fe–S biosynthetic enzyme SufC after challenge with dipyridyl (DP) and paraquat (PQ). Cultures were grown to OD_600_ of 0.8–1.0 and challenged with stressors for 30 min. Cells were harvested, lysed. Clear extracts (50 *μ*g of protein) were subjected to SDS-PAGE and further analyzed by western blot. All western blot assays were performed in triplicate of three independent growth experiments. A representative image from western blot containing two separate gels of wild type and *ΔbshA* exposed in the same film is shown.

In *B. subtilis*, the SUF system encoded by the *sufCDSUB* operon, is proposed to be the only Fe–S cluster biosynthesis machinery (Albrecht et al. 2010; Selbach et al. 2010; Boyd et al. 2014). The lower levels of Fe–S clusters, judged by the lower activity of Fe–S enzymes, could also be attributed to expression or inefficiency of biosynthetic apparatus in assembling Fe–S clusters in the absence of BSH and/or conditions detrimental to Fe–S metabolism. Therefore, the levels of two biosynthetic components, SufC and SufB, were determined by western blot analysis of lysates of cells cultured in MM. Expression levels of both SufC and SufB in wild type were higher than the mutant strain, whereas the internal control MnmA protein, proposed to participate in 2-thiouridine formation (Black and Dos Santos 2015), showed no significant change (Fig. S4). In *E. coli*, the SUF system is expressed only under oxidative stress or iron starvation to promote Fe–S biogenesis under these conditions (Outten et al. 2004). To verify the changes in expression levels of the *B. subtilis* SUF components under stress conditions, western blot analyses of SufC were conducted in cell lysates of MM cultures challenged with DP and PQ for 30 min. Overall, SufC protein levels were similar across various conditions in the wild-type strain (Fig.7). When the *ΔbshA* strain was challenged to various stresses, the levels of SufC increased to comparable levels to those observed in wild-type cultures (Fig.7). This result suggested that expression of *sufC* although constitutive can respond to iron starvation and oxidative stress, but only when BSH is absent.

## Discussion

Since the discovery of BSH in *Bacillus* species in 2009, this biothiol has been proposed to be analogous to GSH and MSH in *B. subtilis* based on its similar structure and dominant concentration (Newton et al. 2009). Subsequent studies identified the involvement of BSH in protecting cells against hypochlorite, diamide, and fosfomycin challenges (Gaballa et al. 2010; Chi et al. 2011, 2013; Posada et al. 2014). However, the stress-related protective mechanisms of BSH as well as other in vivo roles are still being unveiled. Initial growth assays with wild-type *B. subtilis* and *ΔbshA* mutant strains in rich medium, under various conditions, did not show any significant difference (Gaballa et al. 2010). However, *B. subtilis ΔbshA* showed a slow growth phenotype in Spizizen’s MM, which was restored upon addition of GlcNAc-malate and BSSB, supporting the idea that growth defects observed in MM were attributed to lack of BSH. Growth improvement was also observed upon supplementation with selected amino acids (Leu, Ile, Glu, and Gln) or iron. This initial result led us to explore a potential link between BSH and iron metabolism which includes Fe–S cluster biosynthesis. The results showed that deletion of BSH caused severe growth defect under both Cu(I) stress and PQ stress. As early as 1987, it has been reported that Fe–S cluster containing alpha, beta-dihydroxyisovalerate dehydratase in the isoleucine biosynthetic pathway was an target of superoxide (Kuo et al. 1987). Subsequent studies also suggested that Fe–S dehydratases were primary targets of superoxide stress. Fe–S dehydratases can be oxidized at an extremely high rate (3 × 10^6^ mol L^−1^ s^−1^) (Flint et al. 1993), leading to intracellular superoxide overload and consequently a branched-chain amino acid auxotrophy. The investigation of *B. subtilis* described in this study showed that similar oxidative damage appeared to be exacerbated in the absence of BSH, as evidenced by the PQ sensitivity of the mutant strain. In addition to growth inhibition of the Δ*bshA* strain, PQ challenge also resulted in increased levels of intracellular Mn, disulfide pool and BSH-associated to proteins. In combination, these observations highlighted the involvement of BSH in protecting cells against superoxide stress.

Pathways containing Fe–S enzymes are susceptible targets of oxidative stress. Bacterial responses to oxidative stress includes (1) expression of hydrogen peroxide and superoxide scavenging enzymes, (2) repression of Fe- and [Fe–S]-containing enzymes with concomitant expression of enzymes utilizing alternate cofactors, (3) mismetallation of enzyme’s Fe-binding site for Mn or Zn (Imlay 2006; Helmann 2014), and (4) upregulation of Fe–S biosynthetic pathways. In *E. coli*, the two Fe–S cluster biosynthetic machineries ISC and SUF use distinct regulatory strategies to respond to these environmental challenges (Outten et al. 2004). The housekeeping ISC system supplies Fe–S clusters via its Fe–S cluster scaffold IscU. However, under conditions of oxidative stress, transient clusters associated to IscU are also prone to damage thus lowering the efficiency of this system (Jang and Imlay 2010). Instead, under oxidative stress and Fe starvation conditions, Fe–S cluster biogenesis is accomplished by the SUF system. In fact *E. coli* strains lacking SUF components are unable to grow in the presence of DP and are sensitive to PQ and hydrogen peroxide (Outten et al. 2004).

*Bacillus subtilis* and other gram-positive bacteria lack the ISC pathway yet possess a modified SUF pathway which is thought to be the sole pathway for synthesis of Fe–S clusters in this group of bacteria. In fact, genes encoding for the SUF components in *B. subtilis* are essential for survival (Kobayashi et al. 2003; Albrecht et al. 2010). Therefore, we have investigated if expression of the housekeeping SUF components would also be subjected to regulation during iron starvation or oxidative stress. Unlike the expression profile observed for *E. coli*, SufC protein levels did not change significantly with oxidative challenge, suggesting that the *B. subtilis* SUF pathway was being constantly expressed. Our results are in agreement with a recent transcriptomic study in *B. subtilis* cultured under 144 different conditions, which identified the *suf* genes among a small subset of coding sequences (<3% of total genome) that were always expressed at levels higher than the chromosome median (Nicolas et al. 2012). While this study indicated high levels of *suf* genes expression, it did not reveal conditions under which expression was further augmented. Together, these observations provide additional support for the involvement of the *B. subtilis* SUF system in the essential housekeeping formation of Fe–S clusters, a role that is different from the specialized function of the *E. coli* SUF pathway that is only expressed under stress conditions. The decreased activity of Fe–S enzymes in cell lysates lacking BSH under MM growth conditions indicates the potential involvement of BSH in de the novo biosynthesis of Fe–S clusters. Unexpectedly, depletion of BSH caused a twofold reduction in SufC accumulation. It is possible that the lack of BSH induces metabolic responses to protect the cell from oxidative damage. It has been reported that bacterial responses to oxidative damage leads to down regulation of Fe- and Fe–S enzymes with concomitant expression of enzymes utilizing alternative cofactors. The increased levels of Mn-SOD in the Δ*bshA* strain in the absence of any stressor suggested that cells lacking BSH are responding to stress even under standard growth conditions (e.g., minimal medium) and the lower expression of Fe–S biosynthetic components could be seen as another evidence of such response. Interestingly, SufC expression levels in Δ*bshA* strain raised to wild-type levels when cultures were challenged with an iron chelator. *Bacillus subtilis* lacks the Fe–S transcriptional regulator IscR (Giel et al. 2013; Mettert and Kiley 2014) and preliminary results described here suggest that this pathway may be subjected to distinct yet-unidentified mechanism(s).

GSH has been proposed to be the major cytoplasmic iron pool based on its binding affinity (Hider and Kong 2011). In *Salmonella enterica,* GSH has been suggested to be a physiological chelator of the labile iron pool (Thorgersen and Downs 2008), which could provide Fe for Fe–S cluster synthesis, export iron, present ferritin with excess iron, and supply iron directly to cytoplasmic enzymes (Andrews et al. 2003). In addition, GSH and BSH have been shown to associate to proteins, causing protection of cysteine residues from irreversible oxidation (Chi et al. 2011, 2013). In fact protein bacillithiolation increases upon copper, cadmium, and PQ exposure. As cysteine residues are typical ligands of metalloclusters, it is possible to consider that lack of BSH may impact Fe–S enzymes at a step prior cluster insertion. Furthermore, the cysteinyl and malyl moieties of BSH are suitable metal ligands (Newton et al. 2009). Although, it is hypothesized that BSH plays an analogous role to GSH in *B. subtilis*, it is plausible to consider that BSH may also participate in direct metal binding and mediate transport of Fe in bacterial species producing this biothiol.

## Supporting Information

**Figure S1.** Growth profiles of *Bacillus subtilis* wild type (square), *ΔbshA* (circle)*,* and *ΔbshC AmyE:Pxyl-bshC* (triangle) strains in LB (black) and Spizizen’s MM plus Leu, Ile, Gln, and Glu (empty). The growth curves shown are representative of at least of three independent experiments. **Figure S2.** Activities of mononuclear iron enzymes threonine dehydrogenase (A) and quercetin 2,3-dioxygenase (B). Activities were determined with wild-type *Bacillus subtilis* (black) and *ΔbshA* strain (gray) cell lysates from MM (filled bar) and MM plus 50 *µ*mol/L of exogenous Fe^2+^ (upright striped bar). All assays were performed in triplicates. The statistical analysis was performed using unpaired *t* test, *P* values were obtained by comparing each sample with the *ΔbshA* without Fe. Comparison of wild-type cultures with and without Fe was not statically significant. NS, not significant, **P* < 0.05, ***P* < 0.01, ****P* < 0.001, *****P* < 0.0001. **Figure S3.** MnSOD activity in the cell lysates of CU1065 (wt) and HB11002 (bshA) before (black) and 30 min after (gray) the treatment of 100 *µ*mol/L paraquat. The cultures were grown to OD_600_ of 1.0 in MM, and samples were taken before and 30 min after the treatment of 100 *µ*mol/L paraquat. Cell pellets were resuspended, lysed, and assayed for superoxide dismutase. The activity was detected through formation of formazan dye using the superoxide dismutase kit from Cayman Chemical, following the manufacture’s protocol. One unit is defined as the amount of enzyme needed to exhibit 50% dismutation of the superoxide radical. Statistical analysis was performed using unpaired *t* test, *P* values were obtained by comparing *ΔbshA* sample with wild-type sample (NS, not significant, ***P* < 0.01). **Figure S4.** Expression of SufB, SufC and MnmA in cell lysates of wild-type and *ΔbshA* strains. Cell lysates were prepared as described in. Each assay was performed in triplicate. For each wild-type and *ΔbshA* cell lysate, 50 *µ*g of protein were loaded into the same gel. The expressions of SufC, SufB, and MnmA were determined from the same cultures. Each blot containing wild-type and *ΔbshA* cell lysates and known concentrations of each respective purified protein were run in the same gel and exposed in the same film. A presentative western blot is shown above. The bands were quantified through ImageJ and the statistical analysis was performed using unpaired *t* test, *P* values were obtained by comparing *ΔbshA* sample with wild-type sample (NS, not significant, **P* < 0.05, ***P* < 0.01, ****P *< 0.001, *****P* < 0.0001).
