# Supplementary material for: Protective role of bacillithiol in superoxide stress and Fe–S metabolism in Bacillus subtilis
Source: Microbiologyopen. 2015 May 18;4(4):616–31. doi: 10.1002/mbo3.267 (PMC4554457; doi:10.1002/mbo3.267)
Supplement: Supplementary file 1 [file mbo30004-0616-sd1.docx]

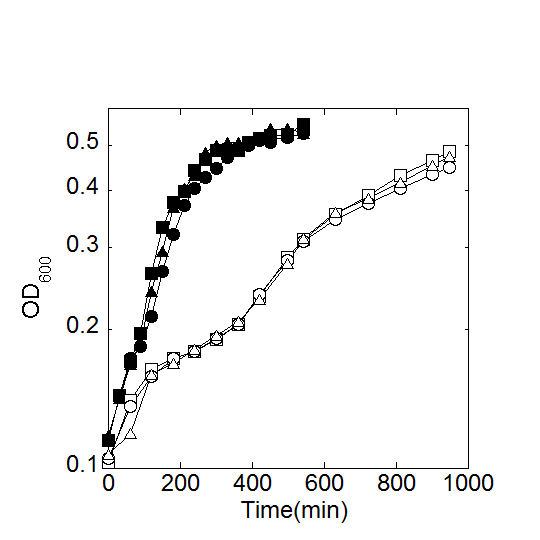


**Figure S1.** Growth profiles of *B. subtilis* wild type (**square**), *∆bshA* (**circle**)*,* and *∆bshC AmyE:Pxyl-bshC* (**triangle**) strains in LB (**black**) and Spizizen’s MM plus Leu, Ile, Gln, and Glu (**empty**). The growth curves shown are representative of at least of three independent experiments.


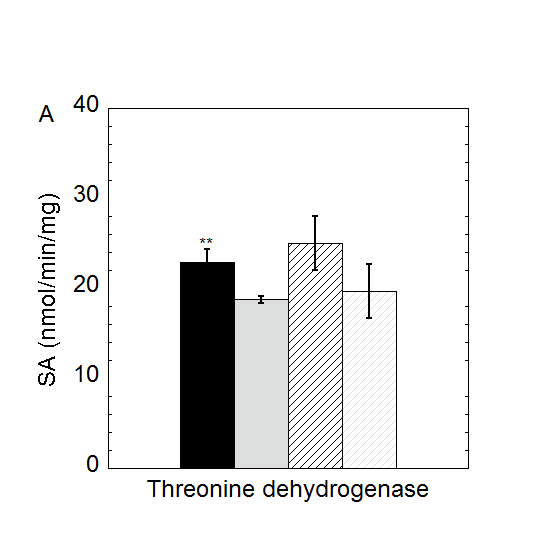

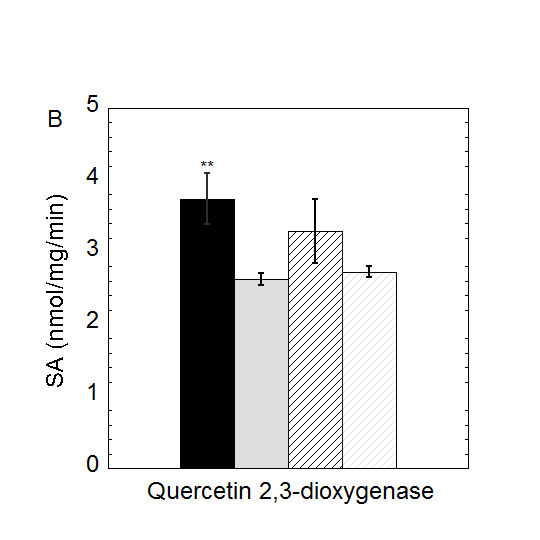


**Figure S2**. Activities of mononuclear iron enzymes threonine dehydrogenase (**A**) and quercetin 2,3-dioxygenase (**B**). Activities were determine with wild type *B. subtilis* (**black**) and *∆bshA* strain (**gray**) cell lysates from MM (**filled bar**) and MM plus 50 µM of exogenous Fe^2+^ (**upright** **striped bar)**. All assays were performed in triplicates. The statistical analysis was performed using unpaired t test, p values were obtained by comparing each sample with the *∆bshA* without Fe. Comparison of wild type cultures with and without Fe were not statically significant. (NS, not significant, p*<0.05, p**<0.01, p***<0.001, p****<0.0001).


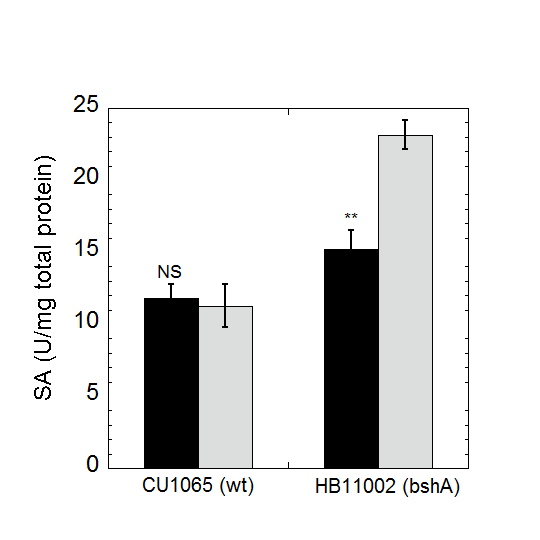


**Figure S3.** MnSOD activity in the cell lysates of CU1065 (wt) and HB11002 (bshA) before (**black**) and 30 min after (**gray**) the treatment of 100 µM paraquat. The cultures were grown to OD_600_ of 1.0 in MM, and samples were taken before and 30 min after the treatment of 100 µM paraquat. Cell pellets were resuspended, lysed and assayed for superoxide dismutase. The activity was detected through formation of formazan dye using the superoxide dismutase kit from Cayman Chemical,following the manufacture’s protocol. One unit is defined as the amount of enzyme needed to exhibit 50% dismutation of the superoxide radical. Statistical analysis was performed using unpaired t test, p values were obtained by comparing *∆bshA* sample with wild-type sample (NS, not significant, p**<0.01).


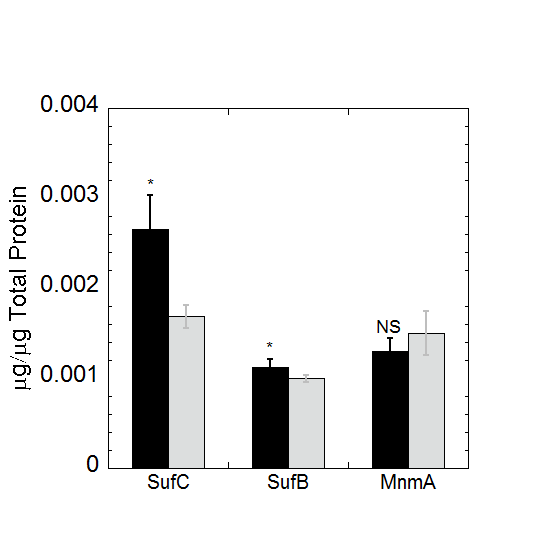


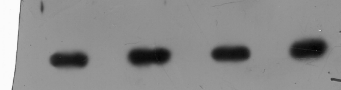

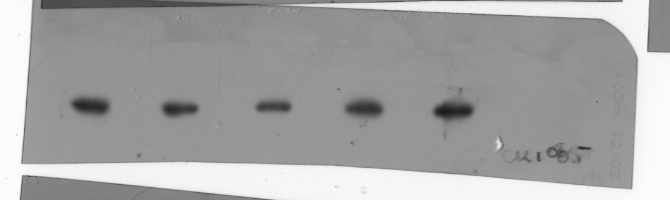

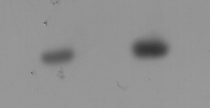


**Figure S4.** Expression of SufB, SufC and MnmA in cell lysates of wild type and *∆bshA* strains*.* Cell lysates were prepared as described in Experimental Procedures. Each assay was performed in triplicate. For each wild type and *∆bshA* cell lysate 50 µg of protein were loaded into the same gel. The expression of SufC, SufB, and MnmA was were determined from the same cultures. Each blot containing wild-type and *∆bshA* cell lysates and known concentrations of each respective purified protein were run in the same gel and exposed in the same film. A presentative western blot is shown above. The bands were quantified through ImageJ and the statistical analysis was performed using unpaired t test, p values were obtained by comparing *∆bshA* sample with wild-type sample (NS, not significant, p*<0.05, p**<0.01, p***<0.001, p****<0.0001).
